# Identifying protein function and functional links based on large-scale co-occurrence patterns

**DOI:** 10.1371/journal.pone.0264765

**Published:** 2022-03-03

**Authors:** Zohar Pasternak, Noam Chapnik, Roy Yosef, Naama M. Kopelman, Edouard Jurkevitch, Elad Segev

**Affiliations:** 1 Division of Identification and Forensic Science, Israel Police, Jerusalem, Israel; 2 Faculty of Management of Technology, Holon Institute of Technology, Holon, Israel; 3 Faculty of Science, Holon Institute of Technology, Holon, Israel; 4 Department of Plant Pathology and Microbiology, The Hebrew University of Jerusalem, Jerusalem, Israel; University of Nebraska-Lincoln, UNITED STATES

## Abstract

**Objective:**

The vast majority of known proteins have not been experimentally tested even at the level of measuring their expression, and the function of many proteins remains unknown. In order to decipher protein function and examine functional associations, we developed "Cliquely", a software tool based on the exploration of co-occurrence patterns.

**Computational model:**

Using a set of more than 23 million proteins divided into 404,947 orthologous clusters, we explored the co-occurrence graph of 4,742 fully sequenced genomes from the three domains of life. Edge weights in this graph represent co-occurrence probabilities. We use the *Bron–Kerbosch* algorithm to detect maximal cliques in this graph, fully-connected subgraphs that represent meaningful biological networks from different functional categories.

**Main results:**

We demonstrate that Cliquely can successfully identify known networks from various pathways, including nitrogen fixation, glycolysis, methanogenesis, mevalonate and ribosome proteins. Identifying the virulence-associated type III secretion system (T3SS) network, Cliquely also added 13 previously uncharacterized novel proteins to the T3SS network, demonstrating the strength of this approach. Cliquely is freely available and open source. Users can employ the tool to explore co-occurrence networks using a protein of interest and a customizable level of stringency, either for the entire dataset or for a one of the three domains—Archaea, Bacteria, or Eukarya.

## Introduction

The next-generation sequencing revolution has resulted in exponentially-growing databases of tens-of-thousands of protein sequences from thousands of organisms. The vast majority of these proteins have not been experimentally tested [1], and experimental characterization of all known proteins is essentially unfeasible [2, 3]. Thus, 72% of the orthologous clusters, or protein families, at the MBGD database were annotated as “hypothetical” or “uncharacterized” [4]. Functional predictions for the majority of the proteins thus rely on computational methods. However, although large scale functional annotation projects have been initiated and many bioinformatics tools have been developed (e.g., [5, 6]), the function of most proteins remains unknown. Based on the assumption that sequence similarity implies functional similarity, conventional computational approaches to protein annotation were based mainly on sequence similarity and the identification of annotated homologs [7]. Sequence similarity can also be used in the context of a network, an approach that has been termed Sequence similarity Network, or SNN [3, 8, 9]. These methods have the weakness of being influenced by the size of the datasets, which has a negative effect on the performance of these tools (e.g. [10]), and, as mentioned above, rely on annotated homologs. Another class of computational methods is based on *3D* protein structures or biophysical features. However, in spite of the progress in utilizing structural approaches [11, 12], these methods require great computational resources, rendering them unsuitable for large datasets. An alternative approach to function prediction is to examine protein interaction networks, and determine proteins’ functions on the basis of the proteins with which they come into physical contact [13]. However, investigating protein-protein interactions is slow and challenging at the experimental level, and direct genome-wide experimentation of functional associations is not feasible in many organisms.

Functional links can also be identified computationally based on the principle of ’*guilt by association’*. The assumption is that if proteins are inherited or lost from a large number of genomes co-dependently, despite multiple evolutionary events of gene loss, speciation and lateral transfer, then they are likely to interact functionally or physically [14]. One of the approaches that are based on this principle is termed *phylogenetic profiling*, a method which is based on the assumption that genes that are functionally related will be characterized by similar presence-absence patterns across different organisms [15, 16]. In light of the different processes that interfere with the patterns of co-occurrence, such as gene loss and lateral transfer, the conservation of a co-occurrence pattern across a large number of species may be taken to indicate functional links between the proteins. Phylogenetic profiling methods have been previously used to predict functional interactions between proteins from the same multi-subunit protein complex or biochemical pathway, to annotate uncharacterized proteins and to discover proteins underlying a specific phenotype [17–19]. Existing phylogenetic profiling tools (e.g [18, 20] generally use a single protein as input, and find its network or clique. These methods have performed well for both eukaryotic and prokaryotic genomes. Some notable recent examples include the identification of novel recombination repair genes [21], the pairing of peptides to GPCR receptors [22], and the identification of secondary metabolic gene clusters [23]. However, a key limitation of these methods is the computational complexity of the related algorithms, and large-scale phylogenetic profiling for eukaryotic species has thus been limited.

Here we present Cliquely, a new phylogenetic profiling tool for protein function prediction. Cliquely uses highly-efficient algorithms, incorporating a dataset of protein sequences obtained from 4,340 prokaryotic, 266 archaeal, and 166 eukaryotic, fully sequenced non-draft genomes. Building on an existing orthology inference from the Microbial Genome Database (MBGD), Cliquely avoids the difficult computational challenge of identifying orthology within thousands of genomes, focusing instead on efficiently utilizing orthology information for the exploration of protein function and conservation, using a network approach. Unlike similar tools [24, 25], Cliquely does not focus on model organisms but on a database encompassing 4,772 species. Generating a protein co-occurrence network based on phylogenetic profiles, Cliquely provides users with a way to explore functional relationships, using a user-defined similarity threshold and fast computations. Supporting single-protein inquiries, Cliquely presents researchers with an option of exploring cliques with respect to the entire dataset, or specifically in the context of Bacteria, Archaea, or Eukaryota. Cliquely thus allows users to explore the co-occurrence networks in the context of different levels of taxonomic resolution, and with unprecedented data in terms of both species and proteins.

## Materials and methods

### Dataset

Fully-sequenced genomes were obtained from the Microbial Genome Database (MBGD, http://mbgd.genome.ad.jp) on 2018. The data contained about 23 million proteins from 4,772 fully sequenced genomes, clustered to 404,947 orthologous groups. Raw data was obtained as a cluster-based, extended-tab archive format, with orthologous clusters generally reflecting protein families. The data associates orthology information, organism identification and functional parameters. Preprocessing consisted of reducing sequence data to a single sequence per cluster chosen at random, and further eliminating unnecessary information as previously described [26, 27]. After preprocessing, the data contained the original 404,947 cluster IDs and the related protein IDs, with a single sequence representing each cluster and the associated functional information.

### Co-occurrence graph

Cliquely operates on presence/absence patterns of clusters, or protein families, across the entire set of organisms at hand. These patterns can be represented as binary vectors of 0’s and 1’s, indicating absence and presence, respectively. These binary vectors are often referred to as *phylogenetic profiles* [15], representing the phylogenetic distribution of orthologous clusters or protein families across organisms. Cliquely operates by generating a co-occurrence graph and quantifying the similarity or correlation between different proteins (or orthologous clusters) through the comparison of their phylogenetic profiles.

Consider a pair of two protein families, or clusters, *u* and *v*. We define a measure, termed *P*_*co*_, which quantifies co-occurrence probabilities, i.e. the probability of presence of one protein cluster given that the other protein cluster is present in the organism. A protein cluster is considered as present in an organism if at least one protein which belongs to the cluster is present in the organism. Formally, for two clusters or protein families *u* and *v*, we define

$$P_{co}(u,\;v)=P(u|v)*P(v|u)$$

In this equation, *P*(*u*|*v*) is the conditional probability for the presence of a protein from cluster *u* in an organism, given that the organism has a protein from cluster *v*. *P*_*co*_ is guaranteed to lie between 0 to 1 and is symmetric with respect to *u* and *v*. It reflects the probability of co-occurrence, with high scores indicating high probability for co-occurrence. An example for *P*_*co*_ calculation in a case of two protein clusters and 10 organisms is given in S2 Text.

**Fig 1:**
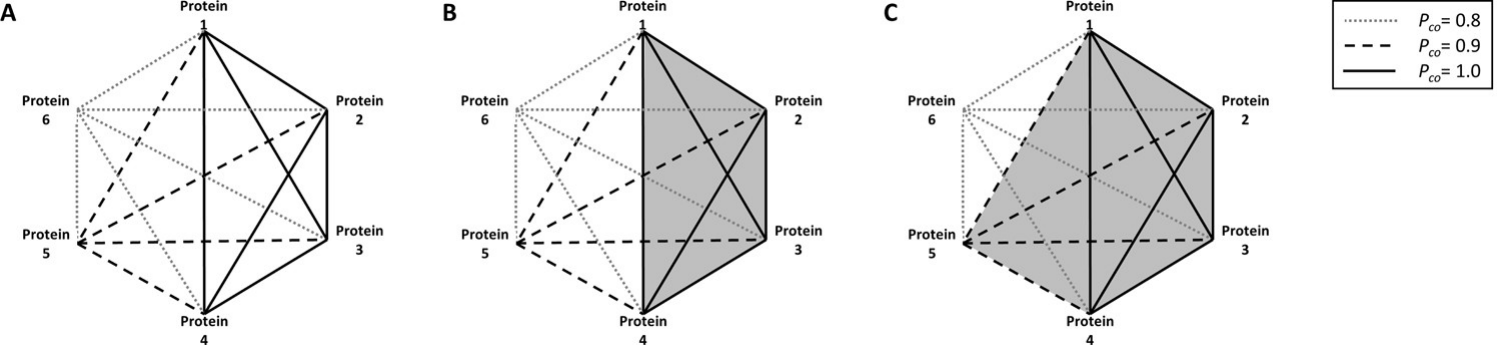
A hypothetical network of 6 proteins with three different cutoffs of *P*_*co*_: *P*_*co*_ = 1 solid line, *P*_*co*_ = 0.9 dashed line, and *P*_*co*_ = 0.8 dotted line. Protein 1 is used as a query. **A.** The graph for the 6 proteins, all edges included. **B.** The maximal Clique identified with *P*_*co*_ = 1 (in gray). The clique contains proteins 1–4. **C.** The maximal Clique identified with *P*_*co*_ = 0.9 (in gray). The Clique contains proteins 1–5.

Cliquley treats orthologous clusters as nodes in a fully connected weighted graph, G = (V, E). Vertices in set V represent clusters or protein families, and *P*_*co*_ scores are used as weights. We expect clusters of proteins that share a biological function or pathway to have similar phylogenetic profiles, and a *P*_*co*_ which is thus close to 1. Similarly, we expect proteins that lie within the same biochemical network to have a *P*_*co*_ close to 1. Given the weighted graph, Cliquely identifies sub-graphs within this graph that are fully connected (i.e., each vertex in the subgraph is connected to all other vertices of the subgraph). Such sub-graphs are also known as *cliques*, and some cliques are defined as *maximal*, i.e., cliques that cannot be further extended. Thus, Cliquely identifies maximal cliques in the graph that contain a protein of interest, or its closest homolog in our dataset. The stringency of the association is determined by a single parameter given by the user, which represents a *P*_*co*_ (i.e. edge-weight) threshold for the inclusion of edges in the graph. Edges whose weights are smaller that this threshold will not be included in the graph. Fig 1 illustrates the influence of this threshold on an identified clique.

The identification of maximal cliques is performed using the Bron–Kerbosch algorithm [28] with pivoting and vertex-ordering. The Bron–Kerbosch algorithm has been shown to be more efficient than other algorithms for sparse graphs, and was used to solve various problems in computational biology (e.g., [29–32]). The worst-case time complexity of this algorithm is O(3^*n*/3^), implying that running times for the full graph could be impractical on a personal computer. Tighter bounds on worst-case running time can be obtained for sparse graphs, depending on the degeneracy of the graph.

Cliquely allows users to examine the properties of co-occurrence graphs, using the user’s protein of interest and a specified co-occurrence probability threshold (*P*_*co*_). To make the process more efficient, the full graph encompassing the entire dataset is first pruned, removing nodes and edges that are of no relevance to the entry (input) protein, and edges with weights that are smaller than the chosen threshold (*P*_*co*_). Graph pruning is performed as a four-step process; Ignoring non-relevant organisms, Cliquely first aggregates protein families (i.e. clusters) from organisms that have at least one member in the protein family (i.e. cluster) associated with the entry protein. Secondly, using the user-specified *P*_*co*_ threshold, protein families (nodes) appearing in a fraction of the organisms that is smaller than *P*_*co*_ are removed. The reduced set of protein families are then used as nodes in a preliminary graph. At the final stage of graph construction, the preliminary graph is further pruned, removing edges whose weights are below the given *P*_*co*_ threshold. Following graph pruning, the resulting graph is then passed to the Bron–Kerbosch algorithm to identify maximal cliques. The results are presented to the user both on the screen and as a text file. Users can then further explore the output cliques, which may correspond to know or novel protein pathways and networks.

### Software & user interface

**Fig 2:**
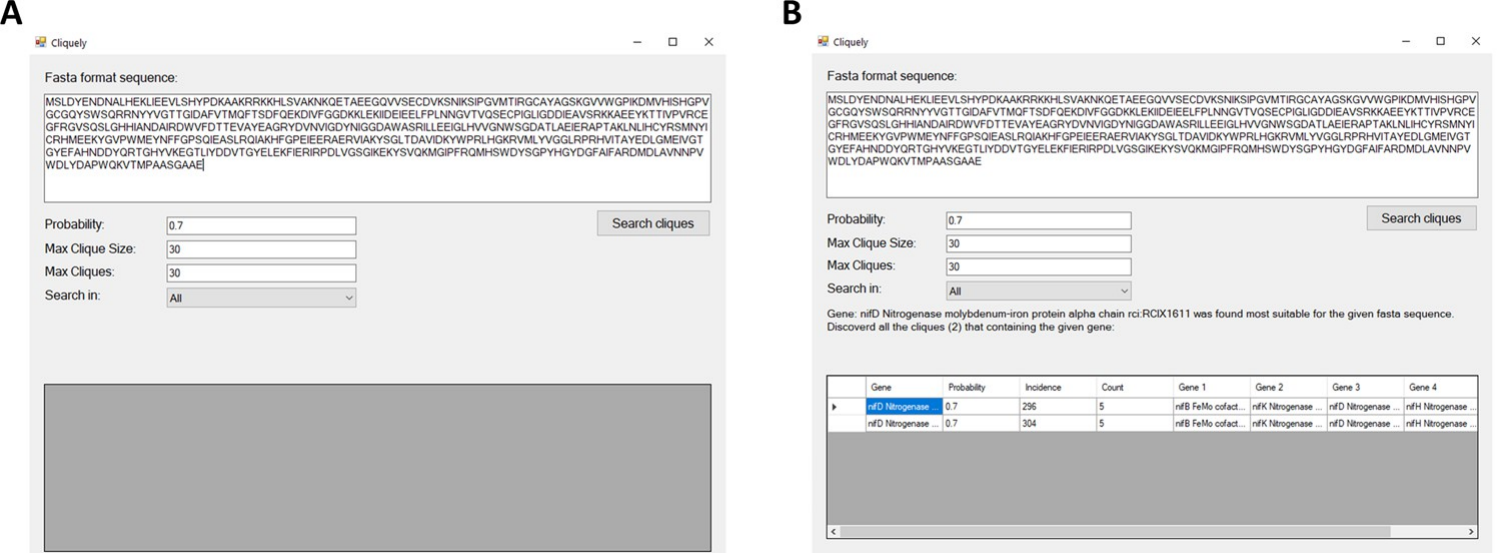
Cliquely’s user interface. **A.** The input window, which allows users to specify a sequence of interest, the parameter of *probability* indicating an edge-weight threshold, the maximal number of cliques and maximal clique size, and a dataset of reference (All, Archaea, Bacteria, or Eukarya). **B.** The output obtained for the example shown in A (two cliques were identified).

Cliquely is designed to operate on the weighted graph described above, which is based on our dataset. Construction of the full graph is computationally demanding, and is thus restricted to incremental updates. Assuming a pre-constructed graph, network exploration is based on single-protein queries, and allows for the identification and exploration of cliques that may represent pathways or molecular complexes, and can thus reveal the functionality of the protein. Users are offered a Windows-compatible downloadable software. Cliquely’s user interface is user-friendly and straightforward (Fig 2). The graphical interface allows users to enter a query protein in FASTA format, specify an edge-weight cutoff (*P*_*co*_, probability of co-occurrence), and define the maximal size and maximal number of cliques to be presented (Fig 1A). In addition, users can choose whether to explore a graph which is based on the entire dataset, or limit their search to either Archaea, Bacteria, or Eukarya. The specified edge-weight cutoff should lie between 0 and 1, and is used in order to prune the adjacency matrix of the graph. An example for how edge-weights are calculated is given in the S2 Text. Graph pruning reduces run time and memory requirements, and filters cliques that are not reliable. The output is presented to the user on screen (Fig 1B), listing identified cliques in separate lines. Cliques are also printed to a csv file for future usage. The code behind Cliquely is freeware, and can be obtained on Github (https://github.com/NoamAndRoy/Cliquely), along with a user manual and running examples.

## Results

### Number of (maximal) cliques and clique size distribution

**Fig 3:**
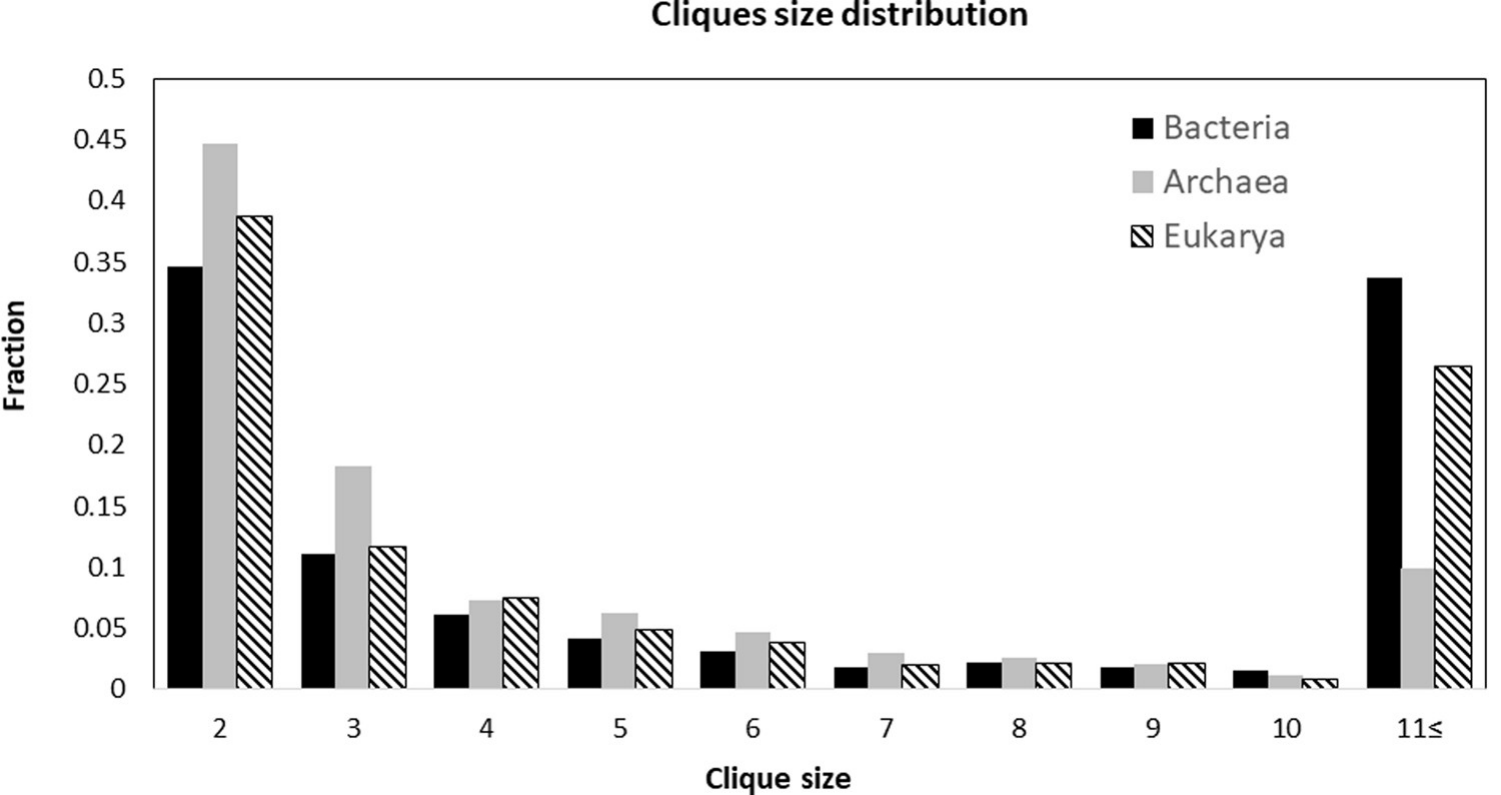
Maximal cliques’ size distributions. Size distributions were examined separately for Archaea, Bacteria and Eukarya, using a threshold of *P*_*co*_ = 1. Cliques of size >11 were binned together.

To illustrate the application of Cliquely, we explored co-occurrence graphs for the entire dataset, consisting of a set of 4,772 fully sequenced non-draft genomes, and more than 23 million proteins divided into 404,947 orthologous clusters. of cliques that may represent pathways or molecular complexes. The properties of maximal cliques were examined across the entire graph as well as separately for the three domains—Archaea, Bacteria, and Eukarya—using a threshold of *P*_*co*_ = 1. The list of all cliques for *P*_*co*_ = 1 can be downloaded from https://github.com/Cliquely/Cliquely. The median maximal clique size was 3 for Archaea, 4 for Bacteria, and 3 for Eukarya. The means vary considerably between the three groups, 5.85±10.05, 56.21±133.56, and 155.5±557.10 for Archaea, Bacteria, and Eukarya, respectively. Due to the differences stemming from larger (maximal) cliques, we also examined maximal clique size distributions specifically for maximal cliques of size 2 to 10 (Fig 3), binning together cliques of size >11. Focusing on this range (2 to 10), the means were 3.39±1.97, 3.43±2.1, and 3.36±1.98, for Archaea, Bacteria, and Eukarya, respectively. Within this range of maximal cliques, the obtained distributions were compared using t-test and found to be similar, although Archaea show higher rates of small maximal cliques and lower rates of large (>11) maximal cliques (Fig 3).

The results presented in Fig 3 illustrate that the distributions of maximal clique sizes for Eukarya and Bacteria are more similar to each other than to the distribution for Archaea. The same trends were observed for lower thresholds of *P*_*co*_ (results not shown).

**Fig 4:**
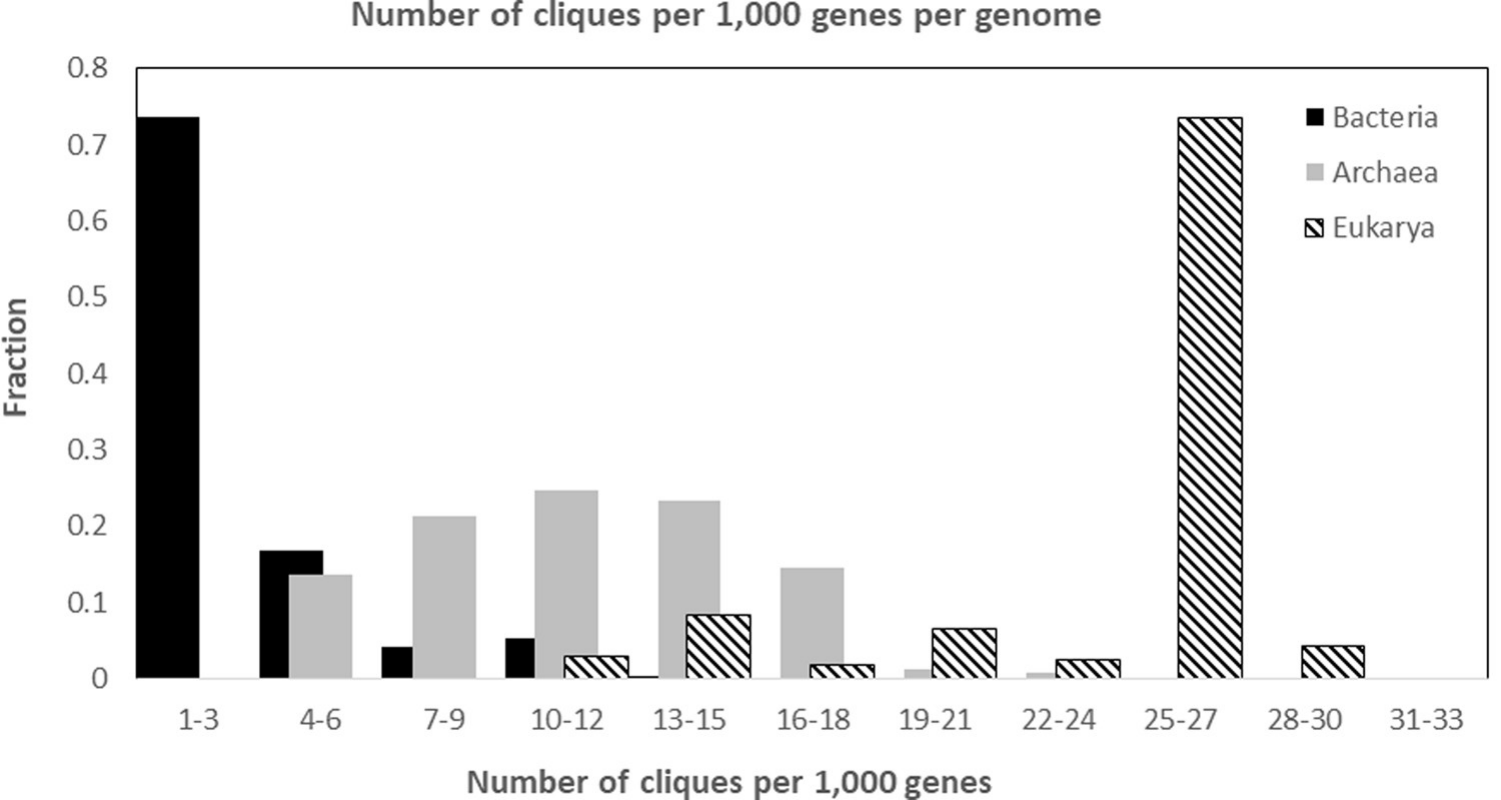
Number of cliques per genome per 1,000 genes. The number of cliques in each genome was normalized per 1,000 genes, to control for genome size differences between the three domains.

We also examined the overall number of cliques per genome, again separately for each domain. Unlike the distributions of clique sizes, the distributions of the number of cliques show remarkable differences between the three domains. Bacteria generally have less cliques per genome than Archaea, and Archaea have less cliques per genome than Eukarya. The average numbers of cliques per genome is 6.60±6.84 for Bacteria, 24.49±10.58 for Archaea, and 124.4±30.01 for Eukarya, with medians of 4, 23, and 126, respectively. A two-sided t-test demonstrated that the three groups are significantly different from each other (p-value of 1.88*10–68 for Bacteria vs. Archaea, 5.9*10–103 for Bacteria vs. Eukarya, and 1.6*10–98 for Archaea vs. Eukarya). To control for differences that stem from differential genome size, we also examined the number of cliques normalized to genome size (Fig 4, presenting the number of cliques per 1000 genes per genome). Overall, the complexity of Eukarya organisms is demonstrated both by the number of cliques per genome as well as by the larger sizes of the identified cliques.

The variation in genome sizes between the three domains, and the correlation between the number of cliques and the number of proteins per genome is also demonstrated in S1 Fig.

### Validation: Exploring known biological networks with Cliquely

Cliquely was validated by examining known networks from various pathways, such as nitrogen fixation, nitrification, methanogenesis and the mevalonate pathways, and ribosome proteins. Using query proteins from different organisms (Table 1 & S1 Table), we ran Cliquely with a range of possible threshold values for co-occurrence (*P*_*co*_). As can be seen in Table 1, the cliques identified by Cliquely included known proteins from these pathways (Table 1; see also S1–S3 Tables). For example, using as query the sequence of NifD, a protein involved in the nitrogen fixation pathway, Cliquely revealed two cliques of proteins belonging to the nitrogen fixation path, including the proteins NifB, NifK, NifD, NifH, NifE NifN, and NifX. Using MvaD–a protein involved in the mevalonate pathway–as a query, Cliquely identified a clique with three additional proteins, mevalonate kinase, HMG-CoA reductase, and phosphomevalonate kinase, all of which are known to be part of this pathway. Another interesting example is the clique of AmoA, AmoB and AmoC, the three small subunits of the enzyme ammonia monooxygenase, playing an essential role in the nitrogen cycle (Klotz et al. 1997). Cliquely correctly identified all three proteins–and only those proteins–as part of a clique, when presented with the AmoB sequence as a query.

**Table 1:** Validation runs of Cliquely, using a single protein as query. Representative proteins in the network are not necessarily the only ones discovered by Cliquely. Stringency is controlled by the parameter *P*_*co*_. See S1 Table for full outputs of these runs.

Query protein / species	Pathway	*P*_*co*_ threshold	No. of cliques	Genomes per clique	Proteins per clique	Representative proteins in clique
NifD /	Nitrogen fixation	0.65	2	262	5	NifB, NifK, NifD, NifH, NifE
*Methanocella arvoryzae*						
				236	3	NIFD, NifN, NifX
MvaD /	Mevalonate	0.65	1	811	3	Mevalonate kinase, HMG-CoA reductase Phosphomevalonate kinase
*Acidianus hospitalis*						
AmoB /	Nitrification	0.6	1	18	3	AmoA, AmoC
*Cenarchaeum symbiosum*						
McrA / *Methanosphaera stadtmanae*	Methano-genesis	1	1	82	12	McrB, McrC, McrD, MMP5, MMP6, MMP7
L10 / *Acidilobus saccharovorans*	Ribosome	1	1	392	7	L18, L32, L15, L30, S8

Further accuracy analyses were carried out for the bacterial chemotaxis pathway. These analyses demonstrate that accurate results can be achieved for range of *P*_*co*_ thresholds (ranging from 0.6 to 0.85) efficiently (see S1 Text, S2 and S3 Tables) with an observable negative correlation between *P*_*co*_ and the number of cliques. Thus, low cutoffs result in the identification of larger cliques. See S1 Text.

### Exploring new horizons: The type III secretion system

Following validation, we examined cliques identified by Cliquely at various thresholds (1, 0.9 and 0.7) for the entire graph (i.e., with no entry protein). As demonstrated in the previous section, many of the Cliquely’s identified cliques correspond to known networks and pathways (glycolysis, ribosome, etc., see Table 1), but several cliques identified on the full graph contained mostly uncharacterized proteins. We examined some of these less-characterized networks, to illustrate the utility of Cliquely.

One of the identified cliques for *P*_*co*_ = 1 contained 46 proteins that perfectly co-occurred in 136 genomes, i.e. whenever one of the network’s proteins was present, all the other 45 proteins were present as well, and whenever one of the proteins was absent, all the others were absent as well. This clique was ubiquitous in–and unique to–the bacterial family Chlamydiaceae, which includes two genera: *Chlamydia* and *Chlamydophila*. Six of the clique’s proteins were known to be connected to the type III secretion system (T3SS), including two structural proteins, FliF and FliH, forming parts of the injectisome [33, 34]. Of the remaining 39 clique proteins, 33 were uncharacterized (i.e. hypothetical). In order to validate the novel T3SS functional assignment of these proteins, the clique was assessed by three different bioinformatic tools that discover T3SS effectors by comparing their sequences with a sequence database of known T3SS effectors. Combined, these tools confirmed 35 of the 46 proteins to be T3SS (Table 2). By using one of the known T3SS proteins from the clique (RT28_RS03915 from *Chlamydia avium*) as query, we additionally compared Cliquely to two more tools: another phylogenetic profiling software called ProtPhylo [18], and the software STRING, that identifies networks based on chromosomal proximity and RNA co-expression [35]. ProtPhylo discovered 39 of the 46 proteins as a network, whereas STRING discovered none. A search of the EffectiveDB database (https://effectors.csb.univie.ac.at) found that different *Chlamydia* strains contain a total of between 100–300 T3SS effector proteins in their genomes. Additional validation of Cliquely is presented in S2 and S3 Tables.

**Table 2:** Comparison of network discovery and functional assignment tools using phylogenetic profiling, T3SS sequence recognition, chromosomal proximity (pro) and coexpression (coe). Tools used were used: EffectiveT3 (http://effectors.org), BPBAac (http://biocomputer.bio.cuhk.edu.hk/T3DB/BPBAac.php), BEAN (http://systbio.cau.edu.cn/bean/index.php), STRING (https://string-db.org), ProtPhylo (http://www.protphylo.org/Phylogenetic.php).

		Phylogen. profiling		Sequence recognition			Pro coe
*Chlamydia avium* locus tag	NCBI annotation	Cliquely	ProtPhylo	EffectiveT3	BPBAac	BEAN	STRING
RT28_RS00170	Membrane protein	+	+				
RT28_RS00425	Membrane protein	+	+			+	
RT28_RS00455	Membrane protein	+	+			+	
RT28_RS00505	Membrane protein	+	+	+		+	
RT28_RS00510	Membrane protein	+	+				
RT28_RS00565	Hypothetical protein	+					
RT28_RS00700	Divalent regulator A	+	+				
RT28_RS00735	Hypothetical protein	+	+	+		+	
RT28_RS00740	Membrane protein	+	+	+		+	
RT28_RS02550	Hypothetical protein	+	+	+			
RT28_RS01210	Late transcription unit B	+	+				
RT28_RS01400	Crp/Fnr transcrip. regulator	+					
RT28_RS01430	Membrane protein	+	+	+		+	
RT28_RS01580	Hypothetical protein	+	+	+		+	
RT28_RS01585	Hypothetical protein	+	+	+		+	
RT28_RS01760	Membrane protein	+		+		+	
RT28_RS01895	Membrane protein	+	+	+		+	
RT28_RS02010	Membrane protein	+	+			+	
RT28_RS02110	Hypothetical protein	+	+	+	+		
RT28_RS02315	Hypothetical protein	+				+	
RT28_RS02465	T3S low calcium chaperone	+	+	+	+	+	
RT28_RS02480	T3SS translocator CopD2	+	+	+	+	+	
RT28_RS02555	Hypothetical protein	+	+	+		+	
RT28_RS02950	Hypothetical protein	+	+			+	
RT28_RS03100	Hypothetical protein	+	+				
RT28_RS03270	Hypothetical protein	+	+				
RT28_RS03915	T3SS effector	+	+				
RT28_RS03300	T3 flagellar biosynthesis	+	+	+	+	+	
RT28_RS03310	Hypothetical protein	+				+	
RT28_RS03330	T3SS effector	+	+			+	
RT28_RS03335	T3SS effector	+	+			+	
RT28_RS03470	Membrane protein	+	+	+		+	
RT28_RS03480	Periplasmic protein	+	+				
RT28_RS03530	Hypothetical protein	+	+			+	
RT28_RS03635	Histone	+	+			+	
RT28_RS03685	Hypothetical protein	+	+	+			
RT28_RS03730	Putative lipoprotein	+	+			+	
RT28_RS03785	Membrane protein	+	+	+		+	
RT28_RS03925	Lipoprotein	+	+				
RT28_RS03950	Hypothetical protein	+					
RT28_RS04015	Hypothetical protein	+	+	+		+	
RT28_RS04125	Membrane protein	+	+			+	
RT28_RS04170	Hypothetical protein	+	+			+	
RT28_RS04300	Hypothetical protein	+	+	+		+	
RT28_RS04435	Hypothetical protein	+	+			+	
RT28_RS04595	Hypothetical protein	+				+	

## Discussion

The vast amount of protein sequence data accumulating in public databases has the potential to improve our understanding on non-model organisms, protein functions and interactions, and more. However, such insights usually are limited by what we already know about protein function. Computational methods such as Cliquely can facilitate this understanding by predicting protein functions for previously unannotated sequences. Cliquely was developed to simplify the task of elucidating protein function, via a straight-forward identification and exploration of co-occurrence patterns and motifs in a co-occurrence graph. Cliquely treats the problem of identifying co-occurrence patterns as a maximal clique problem, and provides users with a practical method for investigating protein function based on co-occurrence. Phylogenetic profiles capture functional constraints, and can be used to explore the functional properties of protein sequences. While other tools are available for investigating protein function, the user-friendly Cliquely revealed protein function for proteins misidentified by other tools, was found to be computationally efficient on a large dataset, and offers a friendly interface. The code of Cliquely is open source, and users have the option of altering the tool to better suit their needs.

Our analysis with Cliquely found that eukaryotic genomes have significantly more cliques than prokaryotic ones, and that the size of these cliques is both higher and more diverse. This can be attributed to the generally larger and more complex genomes of Eukarya, as well as their abundance of pseudogenes. Within the prokaryotes, however, the differences are harder to explain. Archaea and Bacteria share a common gene-dense genome architecture, with about 1000 genes per 1 million basepairs; yet for an unknown reason, Bacteria seem to be much more diverse in terms of rDNA phylogeny and genome size [36]. The range of clique sizes is much higher in Bacteria than in Archaea, which may be explained by their higher overall diversity. On the whole, an archaeal genome possesses about six times more cliques than a bacterial one. It is important to keep in mind that if, for example, a bacterial genome has only one clique, it does not mean that it possesses only a single metabolic pathway or informational processing machinery; what it does mean is that the proteins comprising the other pathways in the genome have diverged—in sequence and/or content—between bacterial strains to such a degree that they are no longer recognized as a single clique. This result is also is in line with the higher diversity and malleability of the bacterial genome, with specializations to specific ecological niches facilitated by high mutation rates [37]. In contrast, mutation in Archaea may be much slower [38], allowing pathways from archaeons in different niches to be more similar than their bacterial counterparts [39]. As for clique size, archaeal genomes have somewhat fewer large cliques (>10 proteins) than bacterial ones. Again, this does not mean that metabolic pathways (for example) in Archaea are smaller or less complex than their bacterial counterparts, only that these pathways may share (on average) less proteins with other strains.

Using a large dataset of more than 23 million protein sequences, we tested the computational efficiency of Cliquely, and validated the obtained biological results. Cliquely identified known networks from various pathways, and revealed the biological function and modality for previously unannotated proteins belonging to the T3SS system, identifying 33 previously uncharacterized proteins as part of this network. This achievement demonstrates one important application of Cliquely: finding new target proteins for antibiotic treatment of infectious diseases. The dramatic rise of antibiotic resistance among bacterial pathogens, which has evolved against nearly every clinically used antibiotic, means that standard antibiotic drugs become less effective. Found in dozens of Gram-negative pathogens, the T3SS virulence factor is an attractive target for novel antimicrobial drugs which will inhibit the pathogen from injecting effector proteins into the cytosol of host cells, thus preventing their ability to cause disease and harm the host [40]. Despite this potential, very few T3SS inhibitors have advanced into clinical trials [41], to some extent due to the fact that new proteins are not easily identifiable as belonging to the T3SS. It is our hope that with Cliquely, large-scale bioinformatic screening of protein co-occurrence networks may help to advance both biological research and clinical applications.

## Supporting information

S1 Fig Number of cliques vs. number of protein families in different genomes. The plot presents the relationship between the number of protein clusters (i.e. gene families) and the number of identified cliques in the genome.
(PDF)

S1 Table Validation runs on Cliquely, using single proteins as queries. We ran Cliquely with a range of possible threshold values of co-occurrence (*P*_*co*_) using different single proteins as queries. Inputs and full outputs are presented. The identified cliques include known proteins from these pathways. *NifD*—the nitrogen fixation pathway. *MvaD*—the mevalonate pathway. *AmoB*—the nitrification pathway. *McrA*—the methanogenesis pathway. *L10*—the Ribosome pathway.
(XLSX)

S2 Table True positives and false positives in Cliquely’s cliques for *CheA*. Compared to KEGG’s bacterial chemotaxis pathway (pathway 02030). *Probability (P*_*co*_*)* is Cliquely’s probability cutoff; Number of proteins is the clique size; the columns *Che*, *MCP*, *Fli* and *Mot* give the number of proteins from each of these groups appearing in the identified clique; *True positive* is the percentage of proteins in Cliquely’s clique that are part of the KEGG pathway. *False positive* is the percentage of proteins in Cliquely’s clique that do not appear in the KEGG pathway. The pathway can be viewed at https://www.genome.jp/pathway/map02030+K03407. The table demonstrate that as *P*_*co*_ decreases, Cliquely identifies more cliques, and the number of proteins per cliques increases (in this example, an average of 3, 9, 16, 18, 29, 30 proteins per clique were identified for *P*_*co*_ values of 0.85, 0.8, 0.75, 0.65, 0.6, respectively). *True positive* percentage decreases with *P*_*co*_ (an average of 100%, 83%, 76%, 77%, 68%, 44% for *Pco* values of 0.85, 0.8, 0.75, 0.65, 0.6, respectively), and false positive percentage increases (an average of 0%, 17%, 24%, 32%, 56% on average for *Pco* values of 0.85, 0.8, 0.75, 0.65, 0.6, respectively). A false positive here does necessarily imply an erroneous identification, but rather that the protein identified by Cliquely as connected to *CheA* does not appear in the small KEGG pathway 02030.
(PDF)

S3 Table ProtPhylo output for *CheA*. *Che* proteins are marked in bold. *CheA* was used as query for the phylogenetic profiling software ProtPhylo (http://ido.helmholtz-muenchen.de/protphylo/Phylogenetic.php), using default parameters. 121 proteins were determined to accompany *CheA*, with increasing Hamming Distance (HD) between the phylogenetic profile of *CheA* and that of a second protein. This distance is in fact the number of organisms (out of 2048 in the ProtPhylo database) for which the presence/absence of the two proteins does not agree. Smaller Hamming Distance implies a more similar evolutionary history. As an example, in 108 out of 2048 organisms *CheW* does not share the presence/absence pattern of *CheA*. Notably, the two tools operate very differently–while Cliquely identifies groups of proteins (or protein families) that operate together (many-to-many relationship), ProtPhylo examines only one-on-one relationships between the query protein and a second protein. Thus, it is not surprising that Cliquely retrieves the main protein groups in the relevant pathway (bacterial chemotaxis) within the identified cliques, in accordance KEGG map 02030 (https://www.genome.jp/pathway/map02030+K03407)–while ProtPhylo identifies many more proteins that have a similar phylogenetic profile, but are not necessarily part of the same pathway.
(PDF)

S1 Text The computational efficiency of Cliquely. (PDF)

S2 Text Calculation of *P*_*co*_. (PDF)

## References

[1] The UniProt Consortium. UniProt: a hub for protein information. Nucleic Acids Research. 2015;43: D204–D212. doi: 10.1093/nar/gku989 25348405 PMC4384041

[2] Brown SD, Babbitt PC. Inference of functional properties from large-scale analysis of enzyme superfamilies. Journal of Biological Chemistry. 2012;287: 35–42. doi: 10.1074/jbc.R111.283408 22069325 PMC3249087

[3] Copp JN, Akiva E, Babbitt PC, Tokuriki N. Revealing Unexplored Sequence-Function Space Using Sequence Similarity Networks. Biochemistry. 2018;57: 4651–4662. doi: 10.1021/acs.biochem.8b00473 30052428

[4] Uchiyama I, Mihara M, Nishide H, Chiba H. MBGD update 2015: microbial genome database for flexible ortholog analysis utilizing a diverse set of genomic data. Nucleic Acids Research. 2014;43: D270–D276. doi: 10.1093/nar/gku1152 25398900 PMC4383954

[5] Koskinen P, Törönen P, Nokso-Koivisto J, Holm L. PANNZER: high-throughput functional annotation of uncharacterized proteins in an error-prone environment. Bioinformatics. 2015;31: 1544–1552. doi: 10.1093/bioinformatics/btu851 25653249

[6] Casimiro-Soriguer CS, Muñoz-Mérida A, Pérez-Pulido AJ. Sma3s: A universal tool for easy functional annotation of proteomes and transcriptomes. Proteomics. 2017;17: 1700071. doi: 10.1002/pmic.201700071 28544705

[7] Kulmanov M, Hoehndorf R. DeepGOPlus: improved protein function prediction from sequence. Cowen L, editor. Bioinformatics. 2019; btz595. doi: 10.1093/bioinformatics/btz595 34009304 PMC8150137

[8] Atkinson HJ, Morris JH, Ferrin TE, Babbitt PC. Using Sequence Similarity Networks for Visualization of Relationships Across Diverse Protein Superfamilies. Jordan IK, editor. PLoS ONE. 2009;4: e4345. doi: 10.1371/journal.pone.0004345 19190775 PMC2631154

[9] Copp JN, Anderson DW, Akiva E, Babbitt PC, Tokuriki N. Chapter Twelve—Exploring the sequence, function, and evolutionary space of protein superfamilies using sequence similarity networks and phylogenetic reconstructions. In: Palfey BA, editor. New Approaches for Flavin Catalysis. Academic Press; 2019. pp. 315–347. doi: 10.1016/bs.mie.2019.03.015 31072492

[10] Lavezzo E, Falda M, Fontana P, Bianco L, Toppo S. Enhancing protein function prediction with taxonomic constraints–The Argot2.5 web server. Methods. 2016;93: 15–23. doi: 10.1016/j.ymeth.2015.08.021 26318087

[11] Peled S, Leiderman O, Charar R, Efroni G, Shav-Tal Y, Ofran Y. De-novo protein function prediction using DNA binding and RNA binding proteins as a test case. Nature Communications. 2016;7: 13424. doi: 10.1038/ncomms13424 27869118 PMC5121330

[12] Radivojac P, Clark WT, Oron TR, Schnoes AM, Wittkop T, Sokolov A, et al. A large-scale evaluation of computational protein function prediction. Nat Methods. 2013;10: 221–227. doi: 10.1038/nmeth.2340 23353650 PMC3584181

[13] Hou J. New Approaches of Protein Function Prediction from Protein Interaction Networks. New Approaches of Protein Function Prediction from Protein Interaction Networks. 2017. p. 118.

[14] Škunca N, Dessimoz C. Phylogenetic Profiling: How Much Input Data Is Enough? PLOS ONE. 2015;10: e0114701. doi: 10.1371/journal.pone.0114701 25679783 PMC4332489

[15] Pellegrini M, Marcotte EM, Thompson MJ, Eisenberg D, Yeates TO. Assigning protein functions by comparative genome analysis: Protein phylogenetic profiles. Proc Natl Acad Sci USA. 1999;96: 4285. doi: 10.1073/pnas.96.8.4285 10200254 PMC16324

[16] Pagel P, Wong P, Frishman D. A Domain Interaction Map Based on Phylogenetic Profiling. Journal of Molecular Biology. 2004;344: 1331–1346. doi: 10.1016/j.jmb.2004.10.019 15561146

[17] Baughman JM, Perocchi F, Girgis HS, Plovanich M, Belcher-Timme CA, Sancak Y, et al. Integrative genomics identifies MCU as an essential component of the mitochondrial calcium uniporter. Nature. 2011;476: 341–345. doi: 10.1038/nature10234 21685886 PMC3486726

[18] Cheng Y, Perocchi F. ProtPhylo: identification of protein–phenotype and protein–protein functional associations via phylogenetic profiling. Nucleic Acids Res. 2015;43: W160–W168. doi: 10.1093/nar/gkv455 25956654 PMC4489284

[19] Tabach Y, Billi AC, Hayes GD, Newman MA, Zuk O, Gabel H, et al. Identification of small RNA pathway genes using patterns of phylogenetic conservation and divergence. Nature. 2013;493: 694–698. doi: 10.1038/nature11779 23364702 PMC3762460

[20] Cromar GL, Zhao A, Xiong X, Swapna LS, Loughran N, Song H, et al. PhyloPro2.0: a database for the dynamic exploration of phylogenetically conserved proteins and their domain architectures across the Eukarya. Database. 2016;2016. doi: 10.1093/database/baw013 26980519 PMC4792532

[21] Sherill-Rofe D, Rahat D, Findlay S, Mellul A, Guberman I, Braun M, et al. Mapping global and local coevolution across 600 species to identify novel homologous recombination repair genes. Genome Res. 2019;29: 439–448. doi: 10.1101/gr.241414.118 30718334 PMC6396423

[22] Foster SR, Hauser AS, Vedel L, Strachan RT, Huang X-P, Gavin AC, et al. Discovery of Human Signaling Systems: Pairing Peptides to G Protein-Coupled Receptors. Cell. 2019;179: 895–908.e21. doi: 10.1016/j.cell.2019.10.010 31675498 PMC6838683

[23] Krause DJ, Kominek J, Opulente DA, Shen X-X, Zhou X, Langdon QK, et al. Functional and evolutionary characterization of a secondary metabolite gene cluster in budding yeasts. Proc Natl Acad Sci USA. 2018;115: 11030–11035. doi: 10.1073/pnas.1806268115 30297402 PMC6205458

[24] Hu Y, Comjean A, Mohr SE, The FlyBase Consortium, Perrimon N. Gene2Function: An Integrated Online Resource for Gene Function Discovery. G3 Genes|Genomes|Genetics. 2017;7: 2855–2858. doi: 10.1534/g3.117.043885 28663344 PMC5555488

[25] Niu Y, Liu C, Moghimyfiroozabad S, Yang Y, Alavian KN. PrePhyloPro: phylogenetic profile-based prediction of whole proteome linkages. PeerJ. 2017;5: e3712. doi: 10.7717/peerj.3712 28875072 PMC5578374

[26] Pasternak Z, Ben Sasson T, Cohen Y, Segev E, Jurkevitch E. A New Comparative-Genomics Approach for Defining Phenotype-Specific Indicators Reveals Specific Genetic Markers in Predatory Bacteria. Platten M, editor. PLoS ONE. 2015;10: e0142933. doi: 10.1371/journal.pone.0142933 26569499 PMC4646340

[27] Segev E, Pasternak Z, Ben Sasson T, Jurkevitch E, Gonen M. Automatic identification of optimal marker genes for phenotypic and taxonomic groups of microorganisms. Ouzounis CA, editor. PLoS ONE. 2018;13: e0195537. doi: 10.1371/journal.pone.0195537 29718935 PMC5931505

[28] Bron C, Kerbosch J. Algorithm 457: finding all cliques of an undirected graph. Commun ACM. 1973;16: 575–577. doi: 10.1145/362342.362367

[29] Voggenreiter O, Bleuler S, Gruissem W. Exact biclustering algorithm for the analysis of large gene expression data sets. BMC Bioinformatics. 2012;13: A10, 1471-2105-13-S18-A10. doi: 10.1186/1471-2105-13-S18-A10

[30] Eblen JD, Phillips CA, Rogers GL, Langston MA. The maximum clique enumeration problem: algorithms, applications, and implementations. BMC Bioinformatics. 2012;13: S5. doi: 10.1186/1471-2105-13-S10-S5 22759429 PMC3382443

[31] Kose F, Weckwerth W, Linke T, Fiehn O. Visualizing plant metabolomic correlation networks using clique-metabolite matrices. Bioinformatics. 2001;17: 1198–1208. doi: 10.1093/bioinformatics/17.12.1198 11751228

[32] Martin YC, Bures MG, Danaher EA, DeLazzer J, Lico I, Pavlik PA. A fast new approach to pharmacophore mapping and its application to dopaminergic and benzodiazepine agonists. J Computer-Aided Mol Des. 1993;7: 83–102. doi: 10.1007/BF00141577 8097240

[33] Betts-Hampikian HJ, Fields KA. The Chlamydial Type III Secretion Mechanism: Revealing Cracks in a Tough Nut. Front Microbio. 2010;1. doi: 10.3389/fmicb.2010.00114 21738522 PMC3125583

[34] Stone CB, Bulir DC, Gilchrist JD, Toor RK, Mahony JB. Interactions between flagellar and type III secretion proteins in Chlamydia pneumoniae. BMC Microbiol. 2010;10: 18. doi: 10.1186/1471-2180-10-18 20096108 PMC2830194

[35] Franceschini A, Szklarczyk D, Frankild S, Kuhn M, Simonovic M, Roth A, et al. STRING v9.1: protein-protein interaction networks, with increased coverage and integration. Nucleic Acids Research. 2012;41: D808–D815. doi: 10.1093/nar/gks1094 23203871 PMC3531103

[36] Kellner S, Spang A, Offre P, Szöllősi GJ, Petitjean C, Williams TA. Genome size evolution in the Archaea. Robinson NP, editor. Emerging Topics in Life Sciences. 2018;2: 595–605. doi: 10.1042/ETLS20180021 33525826 PMC7289037

[37] Lynch M. Evolution of the mutation rate. Trends in Genetics. 2010;26: 345–352. doi: 10.1016/j.tig.2010.05.003 20594608 PMC2910838

[38] Grogan DW, Carver GT, Drake JW. Genetic fidelity under harsh conditions: Analysis of spontaneous mutation in the thermoacidophilic archaeon Sulfolobus acidocaldarius. Proceedings of the National Academy of Sciences. 2001;98: 7928–7933. doi: 10.1073/pnas.141113098 11427720 PMC35445

[39] Wang L, Wang M, Shi X, Yang J, Qian C, Liu Q, et al. Investigation into archaeal extremophilic lifestyles through comparative proteogenomic analysis. Journal of Biomolecular Structure and Dynamics. 2020; 1–13. doi: 10.1080/07391102.2020.1808531 32820705

[40] Duncan MC, Linington RG, Auerbuch V. Chemical Inhibitors of the Type Three Secretion System: Disarming Bacterial Pathogens. Antimicrob Agents Chemother. 2012;56: 5433–5441. doi: 10.1128/AAC.00975-12 22850518 PMC3486574

[41] Vila J, Moreno-Morales J, Ballesté-Delpierre C. Current landscape in the discovery of novel antibacterial agents. Clinical Microbiology and Infection. 2020;26: 596–603. doi: 10.1016/j.cmi.2019.09.015 31574341

