# Supplementary material for: Identifying protein function and functional links based on large-scale co-occurrence patterns
Source: PLoS One. 2022 Mar 3;17(3):e0264765. doi: 10.1371/journal.pone.0264765 (PMC8893610; doi:10.1371/journal.pone.0264765)
Supplement: S1 Fig — The plot presents the relationship between the number of protein clusters (i.e. gene families) and the number of identified cliques in the genome. (PDF) [file pone.0264765.s001.pdf]

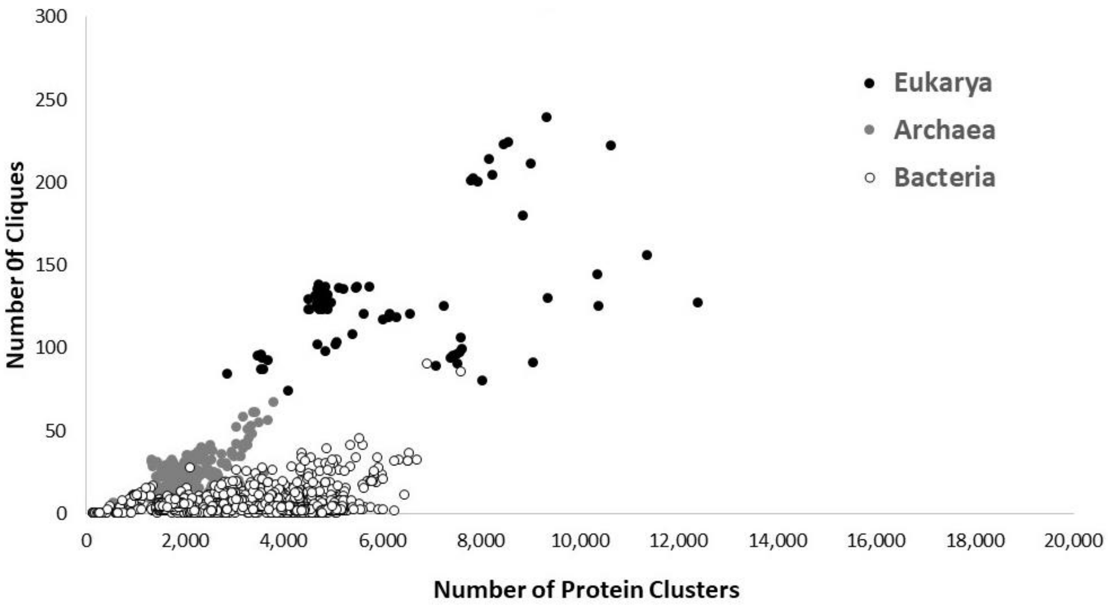


Figure S1. **Number of cliques vs. number of protein families in different genomes.** The relationship between the number of protein clusters (i.e. gene families) and the number of identified cliques in the genome.
