# Supplementary material for: Identifying protein function and functional links based on large-scale co-occurrence patterns
Source: PLoS One. 2022 Mar 3;17(3):e0264765. doi: 10.1371/journal.pone.0264765 (PMC8893610; doi:10.1371/journal.pone.0264765)
Supplement: S2 Table — Compared to KEGG’s bacterial chemotaxis pathway (pathway 02030). Probability (Pco) is Cliquely’s probability cutoff; Number of proteins is the clique size; the columns Che, MCP, Fli and Mot give the number of proteins from each of these groups appearing in the identified clique; True positive is the percentage of proteins in Cliquely’s clique that are part of the KEGG pathway. False positive is the percentage of proteins in Cliquely’s clique that do not appear in the KEGG pathway. The pathway can be viewed at https://www.genome.jp/pathway/map02030+K03407. The table demonstrate that as Pco decreases, Cliquely identifies more cliques, and the number of proteins per cliques increases (in this example, an average of 3, 9, 16, 18, 29, 30 proteins per clique were identified for Pco values of 0.85, 0.8, 0.75, 0.65, 0.6, respectively). True positive percentage decreases with Pco (an average of 100%, 83%, 76%, 77%, 68%, 44% for Pco values of 0.85, 0.8, 0.75, 0.65, 0.6, respectively), and false positive percentage increases (an average of 0%, 17%, 24%, 32%, 56% on average for Pco values of 0.85, 0.8, 0.75, 0.65, 0.6, respectively). A false positive here does necessarily imply an erroneous identification, but rather that the protein identified by Cliquely as connected to CheA does not appear in the small KEGG pathway 02030. (PDF) [file pone.0264765.s003.pdf]

Table S2. True positives and false positives in Cliquely's cliques for CheA compared to KEGG's bacterial chemotaxis pathway (pathway 02030). *Probability (Pco)* is Cliquely's probability cutoff; Number of proteins is the clique size; the columns *Che*, *MCP*, *Fli* and *Mot* give the number of proteins from each of these groups appearing in the identified clique; *True positive* is the percentage of proteins in Cliquely's clique that are part of the KEGG pathway. *False positive* is the percentage of proteins in Cliquely's clique that do not appear in the KEGG pathway. The pathway can be viewed at https://www.genome.jp/pathway/map02030+K03407.

Probability (P_{co})	Number of proteins in the clique	Che	MCP	Fli	Mot	True positive	False positive
0.85	3	1	2	0	0	100%	0%
0.8	12	1	0	6	1	67%	33%
	6	4	2	0	0	100%	0%
0.75	17	2	0	7	2	65%	35%
	22	1	0	11	2	64%	36%
	18	3	1	7	2	72%	28%
	18	4	2	7	1	78%	22%
	5	4	1	0	0	100%	0%
0.7	27	4	2	11	2	70%	30%
	21	3	3	7	2	71%	29%
	8	5	2	0	0	88%	13%
	8	4	3	0	0	88%	13%
	26	3	2	11	2	69%	31%
0.65	30	4	2	11	2	63%	37%
	30	4	3	12	2	70%	30%
	30	5	3	12	2	73%	27%
	29	3	4	12	2	72%	28%
	28	2	2	11	2	61%	39%
0.6	32	1	2	10	3	50%	50%
	30	1	2	8	3	47%	53%
	29	1	2	6	3	41%	59%
	29	1	2	6	3	41%	59%
	28	1	2	5	3	39%	61%

Table S2. True positives and false positives in Cliquely's cliques for CheA compared to KEGG's bacterial chemotaxis pathway (pathway 02030).

The table demonstrate that as *Pco* decreases, Cliquely identifies more cliques, and the number of proteins per cliques increases (in this example, an average of 3, 9, 16, 18, 29, 30 proteins per clique were identified for Pco values of 0.85, 0.8, 0.75, 0.65, 0.6, respectively). True positive percentage decreases with Pco (an average of 100%, 83%, 76%, 77%, 68%, 44% for Pco values of 0.85, 0.8, 0.75, 0.65, 0.6, respectively), and false positive percentage increases (an average of 0%, 17%, 24%, 32%, 56% on average for Pco values of 0.85, 0.8, 0.75, 0.65, 0.6, respectively). A false positive here does necessarily imply an erroneous identification, but rather that the protein identified by Cliquely as connected to CheA does not appear in the small KEGG pathway 02030.
