# Supplementary material for: Identifying protein function and functional links based on large-scale co-occurrence patterns
Source: PLoS One. 2022 Mar 3;17(3):e0264765. doi: 10.1371/journal.pone.0264765 (PMC8893610; doi:10.1371/journal.pone.0264765)
Supplement: S3 Table — Che proteins are marked in bold. CheA was used as query for the phylogenetic profiling software ProtPhylo (http://ido.helmholtz-muenchen.de/protphylo/Phylogenetic.php), using default parameters. 121 proteins were determined to accompany CheA, with increasing Hamming Distance (HD) between the phylogenetic profile of CheA and that of a second protein. This distance is in fact the number of organisms (out of 2048 in the ProtPhylo database) for which the presence/absence of the two proteins does not agree. Smaller Hamming Distance implies a more similar evolutionary history. As an example, in 108 out of 2048 organisms CheW does not share the presence/absence pattern of CheA. Notably, the two tools operate very differently–while Cliquely identifies groups of proteins (or protein families) that operate together (many-to-many relationship), ProtPhylo examines only one-on-one relationships between the query protein and a second protein. Thus, it is not surprising that Cliquely retrieves the main protein groups in the relevant pathway (bacterial chemotaxis) within the identified cliques, in accordance KEGG map 02030 (https://www.genome.jp/pathway/map02030+K03407)–while ProtPhylo identifies many more proteins that have a similar phylogenetic profile, but are not necessarily part of the same pathway. (PDF) [file pone.0264765.s004.pdf]

Table S3: ProtPhylo output for *CheA*. *Che* proteins are marked in bold. CheA was used as query for the phylogenetic profiling software ProtPhylo (http://ido.helmholtz-muenchen.de/protphylo/Phylogenetic.php), using default parameters. 121 proteins were determined to accompany *CheA*, with increasing Hamming Distance (HD) between the phylogenetic profile of *CheA* and that of a second protein. This distance is in fact the number of organisms (out of 2048 in the ProtPhylo database) for which the presence/absence of the two proteins does not agree. Smaller Hamming Distance implies a more similar evolutionary history. As an example, in 108 out of 2048 organisms *CheW* does not share the presence/absence pattern of *CheA*. Notably, the two tools operate very differently – while Cliquely identifies groups of proteins (or protein families) that operate together (many-to-many relationship), ProtPhylo examines only one-on-one relationships between the query protein and a second protein. Thus, it is not surprising that Cliquely retrieves the main protein groups in the relevant pathway (bacterial chemotaxis) within the identified cliques, in accordance KEGG map 02030 (https://www.genome.jp/pathway/map02030+K03407) – while ProtPhylo identifies many more proteins that have a similar phylogenetic profile, but are not necessarily part of the same pathway.

**Description**	**HD**	**Protein**
Chemotaxis histidine kinase (**CheA**)	0	AF1040
Purine-binding chemotaxis protein (**CheW**)	108	AF1044
Protein-glutamate methylesterase (**CheB**)	146	AF1041
Chemotaxis protein methyltransferase (**CheR**)	151	AF1037
Methyl-accepting chemotaxis protein TlpC-1 (PilJ)	326	AF1034
Methyl-accepting chemotaxis protein (TlpC-2)	326	AF1045
glutamine deamidase (**CheD**)	369	AF1038
Chemotaxis response regulator (**CheY**)	494	AF1042
Molybdenum import ATP-binding protein (ModC)	502	AF0092
Molybdopterin-guanine dinucleotide biosynthesis protein A (MobA)	504	AF2005
Site-determining protein	522	AF0696
Ribosomal large subunit 23S rRNA methyltransferase (SpoU)	570	AF1801
NA	583	AF0442
NA	588	AF1397M
UPF0033 protein AF_0188	593	AF0188
UPF0033 protein AF_0556	593	AF0556
Molybdenum cofactor biosynthesis protein (MoaB)	607	AF0265
Uncharacterized protein	607	AF0372
Uncharacterized transporter AF_0510	609	AF0510
NA	612	AF0093
Metalloprotease AF_0655	613	AF0655
NA	613	AF0747
Probable aromatic acid decarboxylase	613	AF1214
NA	614	AF0710
NA	615	AF0933
Cytochrome C-type biogenesis protein (NrfE)	619	AF2192
Nitrogen regulatory protein P-II (GlnB-1)	623	AF0978
Nitrogen regulatory protein P-II (GlnB-2)	623	AF1747
Nitrogen regulatory protein P-II (GlnB-3)	623	AF1750
Molybdenum-pterin-binding protein (MopB)	627	AF1022
Uncharacterized protein AF_0209	632	AF0209
Fumarase (Fum-2)	632	AF1099
Uncharacterized protein	636	AF1039
Uncharacterized protein	642	AF0932
Uncharacterized protein	642	AF1192
Uncharacterized protein	642	AF1211
Uncharacterized protein	642	AF1511
NA	642	AF1837M
Uncharacterized protein	642	AF1938
NA	643	AF0942
Mannose-6-phosphate isomerase/mannose-1-phosphate guanylyl transferase (ManC)	646	AF1097

UPF0097 protein AF_2157	647	AF2157
NA	654	AF2150
Molybdopterin-guanine dinucleotide biosynthesis protein B (MobB)	654	AF2253
NA	654	AF2412
NA	656	AF0219
NA	656	AF0957
NA	656	AF1777
NA	662	AF0909
Alcohol dehydrogenase, iron-containing	666	AF2019
NADH oxidase (NoxA-4)	667	AF0951
Hydrogenase expression/formation protein (HypB)	668	AF1368
Mercuric resistance operon regulatory protein (MerR)	669	AF0673
NA	671	AF1338
Corrinoid methyltransferase protein (MtaC-1)	672	AF0006
Corrinoid methyltransferase protein (MtaC-2)	672	AF0011
NA	672	AF1367
NA	674	AF2265
NA	676	AF0986
Inosine monophosphate dehydrogenase (GuaB-1)	678	AF0847
Uncharacterized protein	678	AF0971
NA	679	AF1677
DNA polymerase, bacteriophage-type	679	AF2277
Nitrate ABC transporter, ATP-binding protein (NrtC-1)	680	AF0087
Nitrate ABC transporter, ATP-binding protein (NrtC-2)	680	AF0638
Phosphate-specific transport system accessory protein PhoU homolog	680	AF1360
Nitrate ABC transporter, permease protein (NrtB-1)	681	AF0086
Nitrate ABC transporter, permease protein (NrtB-2)	681	AF0639
Hydrogenase expression/formation protein (HypC)	681	AF1369
Branched-chain amino acid ABC transporter, ATP-binding protein (BraF-1)	682	AF0222
Ammonium transporter (Amt-2)	682	AF1746
NA	683	AF1516
NA	684	AF1975
Hydrogenase expression/formation protein (HypD)	688	AF1370
Branched-chain amino acid ABC transporter, ATP-binding protein (BraG-2)	689	AF0823
NA	689	AF1390
Signal-transducing histidine kinase	689	AF1483
Hydrogenase expression/formation protein (HypE)	692	AF1365
Hydrogenase expression/formation regulatory protein (HypF)	692	AF1366
Aspartate racemase	694	AF1422
F420-nonreducing hydrogenase (VhtA)	697	AF1380
Uncharacterized protein	697	AF2400
F420-nonreducing hydrogenase (VhtG)	698	AF1381
Uncharacterized MscS family protein AF_1546	698	AF1546
NA	698	AF1950
3-hydroxyacyl-CoA dehydrogenase (Hbd-10)	698	AF2273
NA	699	AF1599

Aspartate aminotransferase (AspB-4)	702	AF0409
Exodeoxyribonuclease III (XthA)	702	AF0580
Branched-chain amino acid ABC transporter, permease protein (BraE-2)	702	AF0824
Branched-chain amino acid ABC transporter, permease protein (BraD-2)	702	AF0825
Branched-chain amino acid ABC transporter, permease protein (BraD-4)	702	AF1392
Methanol dehydrogenase regulatory protein (MoxR)	702	AF2425
Branched-chain amino acid ABC transporter, ATP-binding protein (BraF-2)	703	AF0822
F420-nonreducing hydrogenase (VhtD-2)	703	AF1378
NA	703	AF1389
Peptidyl-prolyl cis-trans isomerase	703	AF1989
Response regulator	706	AF1473
NADH oxidase (NoxB-1)	708	AF0455
NADH oxidase (NoxB-2)	708	AF1262
NA	710	AF1336
Uroporphyrin-III C-methyltransferase (CysG-1)	714	AF0422
Uroporphyrin-III C-methyltransferase (CysG-2)	714	AF1243
NA	715	AF0157
Glutamine ABC transporter, periplasmic glutamine-binding protein (GlnH)	715	AF0231
Molybdopterin oxidoreductase, iron-sulfur binding subunit	715	AF2385
NAD(P)H dehydrogenase (quinone)	716	AF0343
NA	716	AF1280
Uncharacterized protein AF_1281	716	AF1281
Sulfite reductase, desulfoviridin-type subunit gamma (DsvC)	718	AF2228
NA	719	AF1601
Arsenate reductase (ArsC)	720	AF1361
NA	722	AF0949
Phosphate ABC transporter, permease protein (PstA)	722	AF1358
NA	723	AF0061
Iron (III) ABC transporter, permease protein (HemU-1)	723	AF0431
NA	723	AF0629
Iron (III) ABC transporter, permease protein (HemU-2)	723	AF1402
NA	723	AF1761
Conserved hypothetical transmembrane protein	724	AF2388
Aspartate aminotransferase (AspB-1)	725	AF2366

Table S3. ProtPhylo output for *CheA*.
