# Supplementary material for: Identifying protein function and functional links based on large-scale co-occurrence patterns
Source: PLoS One. 2022 Mar 3;17(3):e0264765. doi: 10.1371/journal.pone.0264765 (PMC8893610; doi:10.1371/journal.pone.0264765)
Supplement: S1 Text — (PDF) [file pone.0264765.s005.pdf]

Supplementary text S1 - Computational efficiency

In order to evaluate the computational efficiency of Cliquely, we tested the algorithm using various entry proteins, on a Windows 10 station with Intel(R) Core(TM) i7-6700 CPU @3.40GHz and16GB of RAM, and with the following parameter settings:

max clique size = 100
max cliques = 5

The simulations were carried out for 10 different proteins, each with 9 probability thresholds (P_{co}) : 1, 0.95, 0.9, 0.85, 0.8, 0.75, 0.7, 0.65, 0.6. Thus, a total of 90 simulations were carried out.

Notably, we have terminated simulations lasting over 3 hours, believing that researchers are looking for fast exploration tools, and this will be the main contribution of the software in practice.

The average computation time for the simulations is presented in the following table, by the maximal size of the identified clique, binned into a few categories. Table 1 demonstrates that running time increases as the (maximal) identified clique size increases. However, the running time increases at about X2 (from 18 seconds to 33 seconds) while the maximal clique size increases in an order of magnitude (X10) (comparing the bins of 1-10 proteins in the maximal clique vs. 31-99 proteins in the maximal clique).

Max clique size	#Simulations	Average time (seconds)	SD (seconds)
NA	22		
0	48	18.18	6.67
1-10	6	22.19	6.53
11-30	7	28.56	13.46
31-99	7	33.74	20.25
total	90		

Table 1. The computation time for 90 simulations, binned into categories according to the maximal size of the identified cliques. The NA category includes runs with running time of more than 3 hours. These longer runs were terminated.

In addition, we have also examined the relationship between the Probability parameter (P_{co}) and the computation time. The results are presented in Table 2, and demonstrate that running time increases as P_{co} decreases.

**Probability (P_{co})**	**#Simulations**	**Average time (seconds)**	**SD (seconds)**
NA	22		
1	10	18.28	6.42
0.9/0.95	19	19.35	8.84
0.8/0.85	17	18.57	6.29
0.7/0.75	15	23.03	11.78
0.6/0.65	7	32.88	18.97
total	90		

Table 2. The computation time for 90 simulations, binned into categories according to the probability parameter (P_{co}). The NA category includes runs with running time of more than 3 hours. These longer runs were terminated.
