# Supplementary material for: Identifying protein function and functional links based on large-scale co-occurrence patterns
Source: PLoS One. 2022 Mar 3;17(3):e0264765. doi: 10.1371/journal.pone.0264765 (PMC8893610; doi:10.1371/journal.pone.0264765)
Supplement: S2 Text — (PDF) [file pone.0264765.s006.pdf]

Supplementary text S2 - calculation of P_{co}

Example for the relationship of the proteins across a universe of 10 Bacterial organisms. The table indicates which organisms contain which proteins. For Example, Protein1 is part of the genome of Bacteria1, but is not part of the genome of Bacteria10.

	**Protein1**	**Protein2**
Bacteria1	X	
Bacteria2	X	X
Bacteria3	X	X
Bacteria4	X	
Bacteria5	X	X
Bacteria6		X
Bacteria7	X	X
Bacteria8		X
Bacteria9	X	X
Bacteria10		X

Out of the 8 times Protein2 is present, in 5 times Protein1 is also present; Out of the 7 times Protein1 is present, in 5 times Protein1 is also present, thus

$$P(Protein1|Protein2) = \frac{P(Protein1 \cap Protein2)}{P(Protein2)} = \frac{5}{8}$$

In the in the same way

$$P(Protein2|Protein1) = \frac{P(Protein2 \cap Protein1)}{P(Protein1)} = \frac{5}{7}$$

P_{co} is calculated as the probability of Protein1 to be present in an organism given that Protein2 is present, multiplied by probability of Protein2 to be present in an organism given that Protein1 is present.

$$P_{co}(u, v) = P(u|v) * P(v|u)$$

$$P_{co}(Protein1, Protein2) = P(Protein1|Protein2) \times P(Protein2|Protein1)$$

$$P_{co}(Protein1, Protein2) = \frac{5}{8} \times \frac{5}{7} = 0.45$$

Notably, in our analysis of orthology Protein1 and Protein2 are in fact representatives of a cluster of orthologs. Thus, the co-occurrence that is estimated using by Cliquely using P_{co} is the co-occurrence of representatives of different orthology groups.
